# Molecular mastery: Harnessing DNA technology for disease prevention

**DOI:** 10.1016/j.gendis.2025.101976

**Published:** 2025-12-11

**Authors:** Giri Rajasekhar Dornadula, Ramakrishna Chilakala, Sadak Basha Shaik, Pramod Kumar Meriga, Likhitha Chintha, Yeshwanth Gurugari, Kranthi Kumar D, Sameena Fatima Shaik, Sun Hee Cheong

**Affiliations:** a Department of Pharmacy Practice, Annamacharya College of Pharmacy, Rajampet, Andhra Pradesh 516126, India; b Department of Marine Bio-Food Science, Chonnam National University, Yeosu 59626, Republic of Korea; c College of Dentistry Department of Bio-clinical Sciences, Kuwait University 13110, Kuwait; d Department of Pharmacy Practice, Jawaharlal Nehru Technological University - OTPRI, Ananthapuramu, Andhra Pradesh 515001, India; e Department of Biochemistry, Sri Venkateswara University, Tirupati, Andhra Pradesh 517502, India

**Keywords:** CRISPR-Cas9gene editing, Defective genes, Epigenetic alterations, RNA-based therapies, Somatic cell genome editing

## Abstract

Advances in DNA technology are revolutionizing the landscape of illness prevention, providing new opportunities to combat hereditary abnormalities and chronic diseases before they manifest. CRISPR-Cas9 gene editing, epigenetic alterations, somatic cell genome editing, and RNA-based therapies are all significant advancements in this field. These techniques enable precise changes to DNA and gene expression, potentially preventing a wide range of illnesses at their genetic origins. The following discussion delves into the transformative potential of these technologies. CRISPR-Cas9 is notable for its precision in fixing defective genes, offering promise for avoiding diseases, such as cystic fibrosis and sickle cell anemia. Epigenetic modifications caused by environmental factors are also opening up new avenues for preventing chronic illnesses and malignancies. Furthermore, somatic cell genome editing is a potential, ethically viable strategy as it targets non-reproductive cells, preventing heritable genetic alterations. Along with these developments, RNA-based therapies are emerging as a new avenue for disease prevention. This paper will explore each of these advancements, their applications, and the ethical and technical challenges involved.

## Introduction

The human genome, consisting of roughly 3 billion base pairs, encodes the crucial instructions necessary for regulating cellular processes, development, and physiological balance. The precise coordination of gene expression is essential for maintaining health, while deviations caused by mutations, epigenetic changes, or structural variations can lead to a wide range of diseases, including cancer, cardiovascular disorders, and hereditary syndromes.^1^ These genetic disturbances may be inherited or acquired over time, often initiating pathological changes long before clinical symptoms manifest. Historically, healthcare has concentrated on treating diseases after symptoms appear a reactive model that, although effective in many cases, often falls short in addressing root causes or halting disease progression. In contrast, modern biomedical science now emphasizes a proactive and preventive approach, aiming to identify genetic risk factors early and intervene before pathology develops.^2^ By focusing on the molecular origins of disease, this approach seeks to reduce disease burden and improve long-term health outcomes. Genomic insights have reshaped our understanding of health and disease, highlighting the complex interplay between genetic predisposition, environmental triggers, and lifestyle factors. Integrating this knowledge into preventive strategies allows for risk stratification, early detection, and targeted intervention in at-risk populations.^3^^,^^4^ Consequently, healthcare is transitioning from a one-size-fits-all model to one that is predictive, personalized, and preventive, rooted in the biological uniqueness of each individual.

### Historical context and evolution

The progression of DNA technology has evolved from initial molecular discoveries to advanced therapeutic and diagnostic applications. In the late 1860s, Swiss chemist Friedrich Miescher was the first to identify DNA, which he named “nuclein”.^5^ Almost a century later, the pivotal discovery of the DNA double-helix structure by Watson and Crick in 1953 laid the foundation for modern molecular biology.^6^ The 1970s marked a transformative period with the introduction of recombinant DNA technology, facilitating gene splicing across species and leading to the creation of synthetic insulin and genetically modified organisms.^7^ The 1980s and 1990s were characterized by the development of gene mapping, PCR, and Sanger sequencing, significantly enhancing the ability to identify and study genes associated with diseases.^8^ These technological advancements culminated in the completion of the Human Genome Project in 2003, providing a comprehensive reference of human genes and ushering in the era of personalized medicine.^9^

In the post-genomic era, particularly since 2010, DNA technology has entered a transformative phase. The development of clustered regularly interspaced short palindromic repeats (CRISPR)-CRISPR-associated protein 9 (Cas9) gene-editing technology has enabled precise, efficient, and cost-effective genome editing with wide applications in inherited disease correction, cancer therapy, and infectious disease control.^1^ Simultaneously, the emergence of next-generation sequencing, synthetic biology, RNA-based therapeutics, and epigenome editing has further accelerated advances in precision medicine.

### Next-generation sequencing

Next-generation sequencing, also termed massively parallel sequencing, emerged around 2005–2010 and fundamentally altered genomics by enabling millions of DNA fragments to be sequenced simultaneously at dramatically reduced cost per base.^10^ By 2010, platforms like Roche/454, Illumina, and SOLiD had become commercially available, delivering gigabases of data per run with significantly improved throughput and base accuracy compared with Sanger sequencing.^11^ Next-generation sequencing rapidly transformed applications, such as whole-genome and exome sequencing, transcriptome profiling (RNA sequencing), chromatin immunoprecipitation-sequencing for mapping chromatin states, and cell-free DNA (cfDNA) analysis. Next-generation sequencing platforms enabled population-scale variant discovery (*e.g.*, the 1000 Genomes Project), while reducing sequencing costs by > 100,000 fold in less than a decade.^10^

### Advancement in synthetic biology

**Figure 1:**
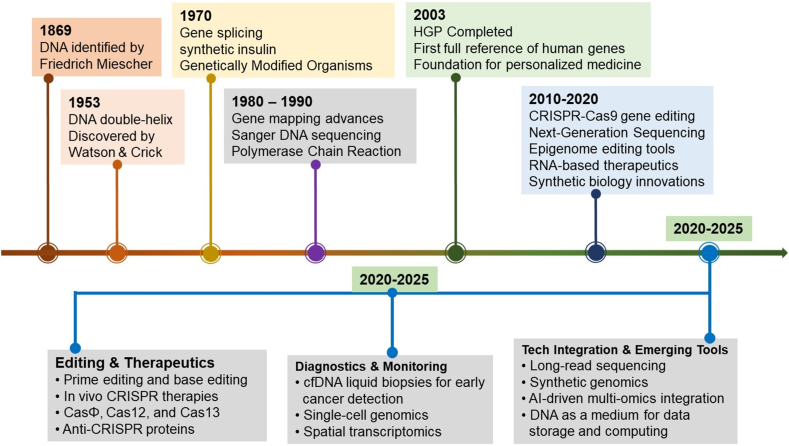
Timeline of major discoveries and milestones in DNA-based disease prevention techniques, from the identification of DNA structure to recent advances in CRISPR-Cas gene editing, mRNA vaccine platforms, and epigenetic biomarker detection.

By 2010, synthetic biology had transitioned from concept to reality. A seminal milestone was the chemical synthesis and transplantation of the Mycoplasma mycoides genome, creating a synthetic organism “Synthia” demonstrating *de novo* genome construction and cellular control.^12^ Review literature of that period highlighted advances in genetic circuit design, such as the toggle switch and repressilator engineered in *E. coli* by Collins, Elowitz, and others, and key foundational work in synthetic gene networks and synthetic biology platforms.^13^ Elowitz and Leibler^14^ had earlier shown that simple engineered networks could yield bistable or oscillatory behavior in living cells, inspiring a wave of design-based biological engineering. By mid-2010, metabolic and regulatory circuit engineering was actively reviewed, with scalable microbial systems for biomanufacturing being reported in Curr Opin Microbiol, emphasizing synthetic biology’s future in industrial applications.^15^ Additionally, improvements in DNA assembly techniques like Gibson Assembly enabled the seamless stitching of multiple large DNA fragments, underpinning whole genome synthesis efforts and synthetic genome construction. The major discoveries and milestones in DNA-based disease prevention techniques, from the identification of DNA structure to recent advances in CRISPR-Cas gene editing, mRNA vaccine platforms, and epigenetic biomarker detection (Fig. 1).^16^

## Recent innovations that have continued to reshape the landscape

### Prime editing and base editing

Prime editing is a next-generation genome editing technology that enables precise and versatile DNA modifications without inducing double-stranded breaks or relying on donor DNA templates. It uses a catalytically impaired Cas9 fused to a reverse transcriptase, along with a prime editing guide RNA (pegRNA), to install small insertions, deletions, and all 12 possible point mutations directly at the target site. This significantly reduces unwanted insertions or chromosomal rearrangements commonly seen with traditional CRISPR-Cas9 methods. Base editing, on the other hand, chemically converts one DNA base into another without cutting the DNA strand, using deaminase enzymes tethered to a modified Cas9. These tools are especially valuable for correcting pathogenic point mutations in monogenic diseases, creating cell and animal models, and potentially for therapeutic genome editing due to their improved precision and safety profiles over earlier genome editing tools.^17^

#### mRNA vaccine technology

The clinical success of mRNA vaccines during the COVID-19 pandemic revolutionized vaccine development by showcasing the platform’s ability to rapidly elicit robust adaptive immune responses. These vaccines use nucleoside-modified mRNA encoding the viral spike protein, encapsulated in lipid nanoparticles for delivery into host cells, where antigen expression triggers protective immune responses. mRNA technology offers key advantages such as speed of development, ease of production, non-integrating properties, and capacity for rapid redesign in response to emerging variants. Beyond infectious diseases, it is being repurposed for personalized cancer vaccines, immunotherapies, and protein replacement strategies in rare genetic disorders, signifying a paradigm shift in preventive and therapeutic medicine.^18^

### Single-cell genomics and spatial transcriptomics

Single-cell genomics and spatial transcriptomics are transformative technologies that allow the profiling of gene expression at single-cell resolution while retaining spatial context within tissues. These methods enable the deconvolution of cellular heterogeneity, identification of rare subpopulations, and mapping of cell-type interactions in complex tissues such as tumors or developing organs. Spatial transcriptomics further allows researchers to localize gene expression patterns within intact histological sections, providing insights into tissue architecture and microenvironmental cues. These tools are indispensable in oncology, developmental biology, and neuroscience for understanding cell differentiation, lineage tracing, and spatial gene regulation.^19^

### Synthetic genomics and genome project-write

Synthetic genomics involves the design and *de novo* synthesis of entire genomes or large genomic constructs, enabling the creation of minimal cells, novel microorganisms, or synthetic eukaryotic chromosomes. The Genome Project-write initiative aims to reduce the cost and increase the scale of writing large genomes, with the goal of building human and non-human genomes from scratch. This technology holds immense promise in xenotransplantation by generating genetically modified organs free of endogenous retroviruses, in microbial production of pharmaceuticals or biofuels, and in developing biosafety features like kill-switches for synthetic organisms. It also raises ethical considerations due to its capacity to rewrite life at a fundamental level.^20^

### CRISPR-Cas12 and Cas13 systems

CRISPR-Cas12 and -Cas13 are novel RNA-guided nucleases with unique properties that expand CRISPR’s applications beyond genome editing. Cas12 targets DNA and exhibits collateral cleavage activity, making it useful for sensitive nucleic acid detection. Cas13 targets RNA and is capable of transcriptome editing without altering the DNA sequence. These enzymes have been adapted into platforms like SHERLOCK and DETECTR for rapid, point-of-care diagnostics of infectious diseases, including COVID-19 and Zika virus. Furthermore, Cas13’s programmable RNA editing opens doors for treating diseases caused by aberrant RNA processing, and Cas12’s robust detection mechanisms have important implications in molecular diagnostics.^21^

#### CasΦ enzyme

CasΦ (Cas-phi) is a compact CRISPR enzyme derived from bacteriophages, notable for its small size and genome editing capabilities. Its hypercompact architecture allows it to be efficiently packaged into delivery vectors such as adeno-associated viruses, overcoming one of the major limitations of conventional CRISPR systems for *in vivo* therapeutic applications. Despite its reduced size, CasΦ retains potent DNA targeting and editing functions, making it a powerful candidate for editing human cells and enabling efficient gene therapy delivery in clinical settings. Its discovery underscores the potential of viral biodiversity in expanding the CRISPR toolbox.^22^

### *In vivo* CRISPR therapies

The development and approval of *in vivo* CRISPR therapies such as exa-cel (formerly CTX001) mark a watershed moment in genomic medicine. Exa-cel employs CRISPR-Cas9 to disrupt the BCL11A enhancer in hematopoietic stem cells, thereby reactivating fetal hemoglobin production in patients with sickle cell disease or β-thalassemia. This autologous *ex vivo* therapy restores effective erythropoiesis and alleviates disease symptoms. Its regulatory approval in multiple regions between 2023 and 2024 demonstrates the clinical viability of CRISPR-based gene editing and has catalyzed further development of genome-editing therapies for various monogenic and acquired disorders.^23^

### Epigenome editing tools

Epigenome editing utilizes nuclease-dead Cas9 (dCas9) fused with epigenetic modifiers to precisely alter gene expression without changing the DNA sequence. This allows for reversible, locus-specific activation or repression of target genes, offering therapeutic strategies for diseases with dysregulated gene expression, such as autoimmune, metabolic, and neurological disorders. By targeting promoters or enhancers, these tools can mimic natural gene regulation and potentially correct disease phenotypes without the risks of permanent genomic alterations. They also facilitate research into epigenetic mechanisms underlying development, cell identity, and disease progression.^24^

### Liquid biopsy and cfDNA

Liquid biopsy involves the analysis of circulating cfDNA in blood for early cancer detection, treatment monitoring, and minimal residual disease assessment. Advanced platforms like Galleri utilize methylation signatures in cfDNA detect multiple cancer types from a single blood sample with high sensitivity and specificity. This non-invasive approach reduces the need for tissue biopsies and allows earlier intervention in asymptomatic individuals. Liquid biopsy is poised to become a cornerstone in personalized oncology, aiding in tumor detection, identifying actionable mutations, and guiding therapy choices.^25^

#### AI-driven genome interpretation and multi-omics integration

AI is increasingly being used to analyze and interpret massive genomic, transcriptomic, proteomic, and metabolomic datasets. Machine learning models can identify pathogenic variants, predict disease risk, and uncover molecular pathways from integrated multi-omics profiles. These tools accelerate biomarker discovery, patient stratification, and drug response prediction, thereby enhancing precision medicine. AI-powered platforms are now essential for converting genomic data into clinically actionable knowledge and are reshaping how healthcare systems implement genomics in diagnostics and therapeutics.^26^

#### Long-read sequencing technologies

Long-read sequencing platforms, such as PacBio Revio and Oxford Nanopore Q20+, have transformed genome sequencing by enabling the resolution of complex structural variants, repetitive regions, and full-length transcript isoforms. These technologies generate contiguous reads exceeding tens of kilobases, allowing for more complete and accurate genome assemblies compared with short-read methods. Their utility spans human genetics, cancer genomics, metagenomics, and transcriptomics, and they are critical for understanding genetic diversity and mapping disease-associated loci in hard-to-sequence regions.^27^

### Anti-CRISPR proteins

Anti-CRISPR proteins are natural inhibitors of CRISPR systems found in bacteriophages and bacteria. They act as molecular off-switches by binding to CRISPR-Cas proteins and preventing their activity, offering a mechanism to control or limit gene editing events. This is particularly valuable for enhancing the safety of CRISPR therapies by reducing off-target effects and allowing temporal control of gene editing. Anti-CRISPR proteins are also being engineered into regulatory circuits and fail-safe mechanisms for therapeutic gene editing and synthetic biology applications.^28^

### DNA-based computing and storage

Programmable DNA computing systems leverage the information-storing capability of nucleic acids to perform logical operations and store vast amounts of digital data. DNA-based logic gates, strand displacement circuits, and molecular memory architectures are being explored for biosensing, secure information storage, and programmable therapeutics. DNA data storage offers extreme density and long-term durability, with potential applications in archiving, encryption, and biosecurity. These technologies represent a convergence of molecular biology and information technology, heralding a new era of biocomputing.^29^

These breakthroughs represent a paradigm shift from reactive to predictive, preventive, and precision-based medicine. As DNA technology continues to advance, it integrates seamlessly with AI, nanotechnology, and systems biology to revolutionize healthcare, agriculture, and synthetic life design.

### Overview of DNA technology in disease prevention

Recent advancements in DNA technologies have markedly transformed disease prevention strategies. These innovations enable the early identification of genetic risks, facilitate the detection of disease susceptibility, and support the implementation of precision interventions tailored to an individual’s genetic profile. Approaches such as genetic screening, polygenic risk scoring, recombinant DNA techniques, gene therapy, CRISPR-Cas9, and RNA-based methods provide comprehensive strategies to prevent disease onset, mitigate complications, and enhance public health outcomes.

### Genetic screening and risk assessment

Genetic screening is a fundamental component of preventive healthcare, as it enables the detection of inherited mutations or genetic variants that increase an individual’s susceptibility to various diseases. By examining specific genes or conducting whole-genome analyses, healthcare professionals can identify pathogenic mutations, such as those in the BRCA1 and BRCA2 genes, which are associated with an increased risk of breast cancer and ovarian cancer. Similarly, mutations in MLH1 and MSH2 are linked to Lynch syndrome, while genetic variants responsible for familial hypercholesterolemia and monogenic diabetes can also be detected.^30^^,^^31^ Identifying these genetic alterations facilitates risk-informed healthcare decisions, including the initiation of enhanced surveillance, chemoprevention strategies, or, in some cases, prophylactic surgical interventions. Furthermore, genetic screening in newborns has been a major public health achievement. Conditions like phenylketonuria and severe combined immunodeficiency, if identified early through newborn screening programs, can be managed effectively, preventing lifelong disability or death.^32^ Thus, genetic screening enables both individual-level disease prevention and population-wide health gains.

### Polygenic risk scores

Polygenic risk scores estimate an individual’s predisposition to complex diseases by aggregating the cumulative effect of numerous genetic variants. Unlike monogenic conditions, common diseases, such as cardiovascular disease, type 2 diabetes, schizophrenia, and breast cancer, often result from the interplay of multiple genes with environmental and lifestyle factors.^33^^,^^34^ Polygenic risk score analysis help clinicians identify high-risk individuals who might otherwise be overlooked by conventional clinical risk models.

Incorporating polygenic risk score analysis into preventive healthcare allows for more accurate stratification of individuals in screening programs and enables targeted interventions, such as lifestyle changes or pharmacological prevention. Although polygenic risk score analysis is still under validation for routine clinical use, expanding biobank databases and improved computational tools are making them more applicable across diverse populations.^35^

### Recombinant DNA technology

Recombinant DNA technology, which involves inserting genetic material from one organism into another, had a transformative impact on both therapeutic and preventive medicine. A major milestone was the production of human insulin using recombinant DNA techniques, which replaced animal-derived insulin and revolutionized diabetes management.^36^ Other applications include the synthesis of clotting factors for hemophilia and growth hormones for growth disorders.

In terms of disease prevention, recombinant DNA technology is foundational in the development of vaccines, such as those for hepatitis B and human papillomavirus, both substantially reduced the global burden of liver cancer and cervical cancer, respectively.^37^ Furthermore, recombinant DNA-based diagnostic tools allow for rapid and accurate detection of infectious agents, making early intervention possible and curbing disease transmission.

### Gene therapy

Gene therapy involves the direct manipulation of genetic material within a patient’s cells to correct, replace, or silence faulty genes responsible for disease. Though initially conceptualized as a curative approach, gene therapy is increasingly being explored for preventive applications, particularly in individuals identified as high-risk before the manifestation of symptoms.^1^^,^^38^ Strategies such as gene addition and editing hold promise for preventing monogenic diseases like spinal muscular atrophy, primary immunodeficiencies, and inherited retinal disorders.

For example, ZOLGENSMA (onasemnogene abeparvovec-xioi), a gene therapy approved for spinal muscular atrophy, exemplifies how early genetic intervention can prevent the progression of severe neurodegeneration. Similar approaches are being developed for hemoglobinopathies and familial cancers, aiming to delay or eliminate disease onset through genetic correction during the preclinical phase.

### CRISPR-Cas9 gene editing

The CRISPR-Cas9 system offers a precise and efficient method for editing specific DNA sequences and holds extraordinary potential in preventing genetic diseases. By enabling targeted correction of pathogenic mutations, CRISPR can theoretically eliminate inherited disorders before they manifest. Clinical applications are being investigated in diseases, such as β-thalassemia, sickle cell disease, and HIV, where editing somatic cells has demonstrated encouraging outcomes.^39^

CRISPR is also being explored for enhancing immune system function, which could protect against infectious diseases or certain cancers. However, its preventive use, especially in germline editing, is met with ethical concerns due to heritability and unknown long-term consequences.^40^ Ongoing research aims to address delivery system challenges, off-target effects, and regulatory barriers before CRISPR can be fully adopted for preventive use in clinical practice.

### RNA-based therapies

RNA-based technologies have emerged as vital tools in preventive medicine, with significant advancements over recent years. One of the most notable successes has been the development of mRNA vaccines, which gained global recognition during the COVID-19 pandemic due to their rapid design, scalability, and high immunogenicity.^41^ These vaccines, created using the genetic sequence of the virus, elicited strong immune responses and played a crucial role in reducing transmission. Building on this success, the mRNA vaccine platform is now being adapted for use in developing vaccines against other diseases, including influenza, HIV, and various cancers.

Beyond vaccine development, RNA-based approaches such as RNA interference and antisense oligonucleotides have shown promise in regulating gene expression. These therapies are under investigation for their potential preventive applications in conditions like transthyretin amyloidosis, hypercholesterolemia, and neurodegenerative disorders.^42^ By specifically targeting and down-regulating genes implicated in disease pathogenesis, RNA-based therapies offer a strategic means of mitigating disease progression at the molecular level.

This review explores the profound impact of DNA-based technologies on disease prevention, highlighting key advancements such as CRISPR-Cas9, epigenetic modifications, somatic cell genome editing, and RNA-based interventions. Additionally, it critically evaluates the challenges and ethical considerations associated with integrating these cutting-edge technologies into clinical practice.

### The power of gene editing: CRISPR-Cas9

CRISPR-Cas9 represents a groundbreaking innovation in molecular biology, offering precise and efficient genetic editing capabilities. Originating from a natural adaptive immune system in bacteria, this technology leverages the CRISPR system in combination with the Cas9 nuclease to specifically target and alter DNA sequences with a high degree of accuracy. Its ability to introduce targeted modifications makes CRISPR-Cas9 a powerful tool for research and therapeutic applications.^39^

### Mechanism

**Figure 2:**
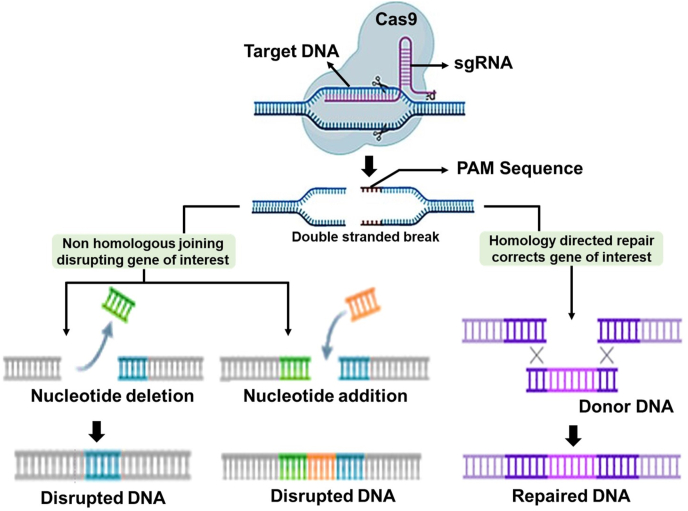
CRISPR-Cas9 gene editing process.

The CRISPR-Cas9 technique relies on two main components: a guide RNA (gRNA) that directs the system to a specific DNA sequence and the Cas9 protein, which creates double-strand breaks at the targeted location. Once the DNA is cleaved, the cell’s natural repair mechanisms take over, enabling the introduction of insertions, deletions, or corrections in the genetic code (Fig. 2).^43^ The applications of correcting genetic mutations are shown in Table 1.

**Table 1:** Applications of CRISPR-Cas9 in correcting genetic mutations.

Gene(s)	Associated diseases(s)	Potential CRISPR-Cas9 application
CFTR	Cystic fibrosis	Correcting mutations in the CFTR gene to restore normal function, potentially offering a permanent solution to cystic fibrosis.
HBB	Sickle cell anemia	Rectifying mutations in the HBB gene to produce normal hemoglobin, aiming to cure sickle cell anemia.^43^
DMD	Duchenne muscular dystrophy	Restoring dystrophin expression in muscle cells by correcting mutations in the DMD gene, highlighting potential treatment for genetic muscular disorders.^44^
CCR5	HIV/AIDS	Altering the CCR5 gene to prevent HIV from entering and infecting human cells, effectively rendering them immune to the virus.^45^^,^^46^
BRCA1, BRCA2	Hereditary breast cancer and ovarian cancer	Modifying these genes to reduce susceptibility to hereditary cancers, offering hope for individuals with a familial predisposition to malignancies.
Multiple genes	Various cancers	Simultaneously targeting multiple genes involved in tumor development, such as tumor suppressor genes and oncogenes, to develop cancer prevention strategies.
Various genes	Infectious diseases	Enhancing resistance to infectious diseases by modifying human microbiota, offering new avenues for preventing infections and modulating immune responses.^47^

### Successes

CRISPR-Cas9 has transformed the field of genetic medicine by enabling precise and efficient genome editing. A major milestone was the approval of exagamglogene autotemcel (Casgevy™) in 2023, the first CRISPR-based therapy approved by the US FDA and UK MHRA for treating sickle cell disease and transfusion-dependent β-thalassemia.^48^ This therapy uses CRISPR to edit hematopoietic stem cells *ex vivo* to induce fetal hemoglobin expression, thereby alleviating symptoms of these hemoglobinopathies. In oncology, CRISPR has shown promising early results in editing T cells to improve their ability to target cancer cells. Ongoing trials are also exploring CRISPR-based interventions in genetic blindness, inherited metabolic disorders, and HIV, demonstrating the technology’s vast therapeutic potential.

### Failures

Despite notable breakthroughs, CRISPR-Cas9 is not without setbacks. One of the most widely criticized events was the creation of gene-edited babies in China in 2018, where CRISPR was used to edit the CCR5 gene in embryos, sparking global outrage and leading to legal consequences for the researchers involved.^49^^,^^50^ On the technical side, unintended off-target edits, limited editing efficiency in certain tissues, and immune responses to Cas9 protein have raised concerns about the platform’s safety and reliability, particularly for *in vivo* applications.

### Challenges

CRISPR-Cas9’s clinical translation faces several scientific and logistical challenges. A primary technical concern is off-target activity, where the Cas9 enzyme may induce double-strand breaks at unintended genomic loci, potentially leading to mutagenesis or oncogenesis. To mitigate this, newer high-fidelity variants like SpCas9-HF1, eSpCas9, Cas12a (Cpf1), and Cas13 have been developed to enhance target specificity and reduce unwanted edits.^51^ Another major challenge is efficient and targeted delivery of the CRISPR components to specific cells or tissues. Current approaches include viral vectors (*e.g.*, adeno-associated viruses, adeno-associated viruses) and non-viral systems (*e.g.*, lipid nanoparticles), but these face limitations in cargo size, immunogenicity, tissue tropism, and production scalability. Additionally, concerns about the long-term stability, cost of personalized cell therapies, and manufacturing complexities continue to hinder widespread clinical implementation.

### Ongoing clinical trials

Several clinical trials are actively evaluating CRISPR-Cas9 for various genetic conditions, notable among them: i) NCT03745287: A trial evaluating CTX001 (now Casgevy) for sickle cell disease, showing promising safety and efficacy data; ii) NCT03872479: A trial investigating EDIT-101, an *in vivo* CRISPR treatment targeting CEP290 mutations in patients with Leber congenital amaurosis type 10 (LCA10), a rare inherited form of blindness. Other trials are exploring CRISPR’s role in cancer immunotherapy, amyloidosis, and HIV, marking an expanding pipeline of therapeutic applications.^52^^,^^53^

### Ethical considerations

Ethical concerns remain a major consideration, especially surrounding germline genome editing. Unlike somatic editing, germline modifications can be inherited by future generations, raising complex questions about consent, unintended long-term consequences, and the risk of misuse for non-therapeutic purposes such as genetic enhancement or eugenics. The 2018 case of gene-edited embryos in China catalyzed global efforts to implement stronger ethical oversight and international consensus on responsible use.^54^ While somatic cell editing is generally accepted when conducted under clinical regulation, germline editing is currently prohibited or heavily restricted in most countries. Ongoing public discourse and the development of international regulatory frameworks are essential to ensure the ethical and equitable use of CRISPR technologies.^55^

### Epigenetic modifications: beyond the genetic code

Epigenetic modifications, which refer to alterations in gene expression without changing the underlying DNA sequence, are essential for regulating cellular functions and the body’s response to environmental influences. These changes are facilitated by mechanisms such as DNA methylation, histone modification, and interactions with non-coding RNAs, all of which control the accessibility and activity of specific genes (Table 2).^56^ Epigenetics adds a flexible layer of regulation that can be influenced by environmental factors, including diet, stress, and exposure to pollutants, positioning it as a crucial focus for disease prevention strategies.

**Table 2:** Mechanism of Epigenetic modifications.

Mechanism	Description	Key enzymes	Impact	Examples in disease prevention
DNA methylation	Addition of a methyl group (-CH_3_) to the 5′ position of cytosine residues in CpG dinucleotides often leads to gene silencing.	DNA methyltransferases (DNMTs): DNMT1, DNMT3A, DNMT3B. Methyl-CpG-binding domain proteins.^57^	Silences gene expression by preventing transcription factor binding and recruiting repressive complexes Critical for genomic imprinting and X-chromosome inactivation^58^	Aberrant hypermethylation of tumor suppressor genes (*e.g.*, *CDKN2A*) in cancers can be reversed by DNMT inhibitors (*e.g.*, azacitidine).
Histone modifications	Post-translational modifications (*e.g.*, acetylation, methylation, phosphorylation) of histone proteins affect chromatin structure and gene expression.	Histone acetyltransferases and histone deacetylases (HDACs). Histone methyltransferases and demethylases	Acetylation loosens chromatin, promoting transcription. Methylation can activate or repress genes depending on the site (*e.g.*, H3K4me activates, H3K27me represses).^59^	HDAC inhibitors (*e.g.*, vorinostat) reactivate silenced tumor suppressor genes. Used in cancer prevention and treatment strategies
Non-coding RNA regulation	ncRNAs regulate gene expression by interacting with mRNAs, DNA, or chromatin.	MicroRNAs (miRNAs): Bind to mRNA to suppress translation or induce degradation. Long non-coding RNAs (lncRNAs): Scaffold proteins to regulate chromatin.	Controls gene silencing and activation through RNA interference mechanisms^56^	miRNAs targeting oncogenic pathways (*e.g.*, miR-34a in p53 activation) are being developed for cancer prevention.
Chromatin remodeling	Chromatin remodeling complexes reposition, eject, or restructure nucleosomes to alter chromatin accessibility.	SWI/SNF complexes ISWI complexes	Exposes DNA for transcription or compacts it for repression Crucial for DNA repair, replication, and transcription	Aberrant remodeling in SWI/SNF genes (*e.g.*, *ARID1A* loss) can lead to cancer. Targeting remodeling pathways is being explored for therapeutic and preventive interventions.
Cross-talk between mechanisms	Epigenetic mechanisms often interact, such as DNA methylation recruiting histone deacetylases to compact chromatin. Non-coding RNAs can guide enzymes to specific sites.	DNMTs, HDACs, miRNAs	Ensures precise gene regulation through coordinated actions^58^	Epigenetic biomarkers like *SEPT9* hypermethylation for early colorectal cancer detection allow personalized prevention strategies.

In addition to the mechanistic roles outlined in Table 2, epigenetic modifications of genes have significant implications for preventive medicine that deserve attention. A notable feature of these modifications is their reversibility, which sets them apart from permanent genetic mutations and presents an opportunity for early interventions before irreversible changes to cellular processes take place. For example, certain dietary factors, such as folate, polyphenols (*e.g.*, curcumin and resveratrol), and sulforaphane, have been found to affect DNA methylation and histone modifications, highlighting the potential for lifestyle and nutritional choices to influence epigenetic regulation.^59^^,^^60^ Moreover, exposure to environmental toxins, tobacco smoke, or stress can lead to detrimental epigenetic alterations, linking external factors directly to disease susceptibility.^61^ These modifiable risks underscore the importance of epigenetic monitoring in early detection and risk stratification for complex diseases, such as cancer, metabolic syndrome, and neurodegenerative disorders.

Another emerging concept in this field is epigenetic memory and transgenerational inheritance, where certain epigenetic marks, especially those induced by environmental exposure, can be passed from parents to offspring, influencing disease risk in future generations.^62^ This insight further strengthens the rationale for preventive strategies at the population level, including maternal and perinatal health interventions. Furthermore, integration of multi-omics technologies (*e.g.*, epigenomics, transcriptomics, and metabolomics) is enabling the identification of dynamic epigenetic biomarkers and their interplay with genetic and metabolic networks, enhancing personalized prevention frameworks. Importantly, clinical trials involving epigenetic drugs are not only targeting treatment but are increasingly being designed with chemoprevention endpoints, particularly in individuals with hereditary cancer syndromes or precancerous lesions.^63^ Therefore, a comprehensive understanding of how epigenetic modifications respond to both intrinsic and extrinsic cues is vital for developing effective, personalized, and population-based disease prevention strategies.

### Applications

One of the most significant applications of epigenetic research lies in its potential to prevent chronic diseases. Abnormal DNA methylation patterns are commonly associated with cancer. Hypermethylation of tumor suppressor genes, such as *CDKN2A* and *MLH1*, has been implicated in colorectal cancer, lung cancer, and breast cancer. By developing drugs that reverse these aberrant methylation patterns, researchers aim to halt the progression of precancerous lesions and reduce cancer incidence.^57^ Histone deacetylase inhibitors, another class of epigenetic drugs, have shown promise in reactivating silenced tumor suppressor genes, further enhancing their utility in cancer prevention.^58^ In addition to cancer, epigenetic modifications are implicated in metabolic and neurodegenerative disorders. Aberrant histone acetylation and DNA methylation patterns have been linked to insulin resistance and type 2 diabetes, offering new avenues for early intervention.^64^ Similarly, hypermethylation of genes associated with neuronal function has been observed in Alzheimer’s disease, suggesting potential targets for epigenetic therapies aimed at preserving cognitive health.

Epigenetics also holds promise in the field of personalized medicine. Epigenetic biomarkers, such as methylation patterns in circulating DNA, can serve as early indicators of disease risk. For instance, hypermethylation of the *SEPT9* gene is a well-established marker for colorectal cancer, enabling early detection and intervention.^56^ Such biomarkers can be used to develop individualized prevention strategies, tailoring lifestyle modifications and pharmacological interventions to an individual’s unique epigenetic profile.

### Successes

Epigenetic therapies targeting DNA methylation and histone acetylation have achieved clinical impact. The hypomethylating agents azacitidine and decitabine inhibit DNA methyltransferases and are approved by the FDA for treating myelodysplastic syndromes and older patients with acute myeloid leukemia. These agents have been shown to improve overall survival and response rates, with azacitidine extending median survival to ∼24 months versus 15 months compared with standard care in patients with high-risk myelodysplastic syndromes. Histone deacetylase inhibitors, notably vorinostat, gained FDA approval for cutaneous T cell lymphoma, marking the first epigenetic therapy targeting histone acetylation in cancer. Moreover, epigenetic biomarkers like SEPT9 promoter hypermethylation serve as early detection markers in colorectal cancer, enabling minimally invasive cancer screening.^64^^,^^65^

### Failures

Despite these advances, first-generation epigenetic drugs suffered from broad activity and limited specificity, impacting gene sets beyond intended targets, often leading to unintended silencing or activation. The transient nature of epigenetic modifications, together with the eventual emergence of drug resistance, limits prolonged therapeutic benefit. Off-target epigenetic changes have also been associated with adverse effects, including cytopenias and secondary hematologic malignancies in long-term use.^66^^,^^67^

### Challenges

Key challenges in epigenetic therapy include the reversible and dynamic state of epigenetic marks making durable gene regulation difficult and distinguishing causative modifications from bystander epigenetic changes in disease. The therapeutic landscape is further complicated by interpatient heterogeneity in methylation and acetylation patterns. Advances in high-throughput sequencing, single-cell epigenomics, and computational modeling are essential to identify clinically relevant targets and predict treatment response.^57^

### Ongoing clinical trials

Current trials are investigating combinatorial regimens to enhance efficacy and reduce resistance. For example, combining azacitidine or decitabine with histone deacetylase inhibitors (*e.g.*, entinostat, vorinostat, panobinostat) has demonstrated preclinical synergy-enhanced tumor cell differentiation, immune signaling, and reduced proliferation in leukemia models. Notable clinical trials include NCT02915523 and NCT02572687, which evaluate the safety and efficacy of these combinations in treating myelodysplastic syndromes, acute myeloid leukemia, and solid tumors.^68^^,^^69^

### Somatic cell genome editing: a targeted approach

Somatic cell genome editing refers to the precise modification of the DNA within somatic (non-reproductive) cells, allowing genetic alterations to be confined to an individual without being passed on to offspring. This technique has emerged as a powerful and ethically permissible strategy to correct disease-causing mutations and enhance therapeutic efficacy in various disorders. The underlying concept involves harnessing site-specific nucleases such as CRISPR-Cas9, zinc finger nucleases (ZFNs), and transcription activator-like effector nucleases (TALENs) to introduce targeted breaks in the genome. These breaks are then repaired by the cell’s intrinsic DNA repair machinery, either via non-homologous end joining (NHEJ), which introduces insertions or deletions that disrupt gene function, or homology-directed repair (HDR), which enables precise gene correction when a donor template is present.^70^ Somatic editing holds significant therapeutic promise because it circumvents the controversial implications of germline editing while addressing a wide spectrum of genetic disorders at their source.^44^

## Types and mechanism of somatic cell editing

### *In vivo* somatic cell genome editing

**Figure 3:**
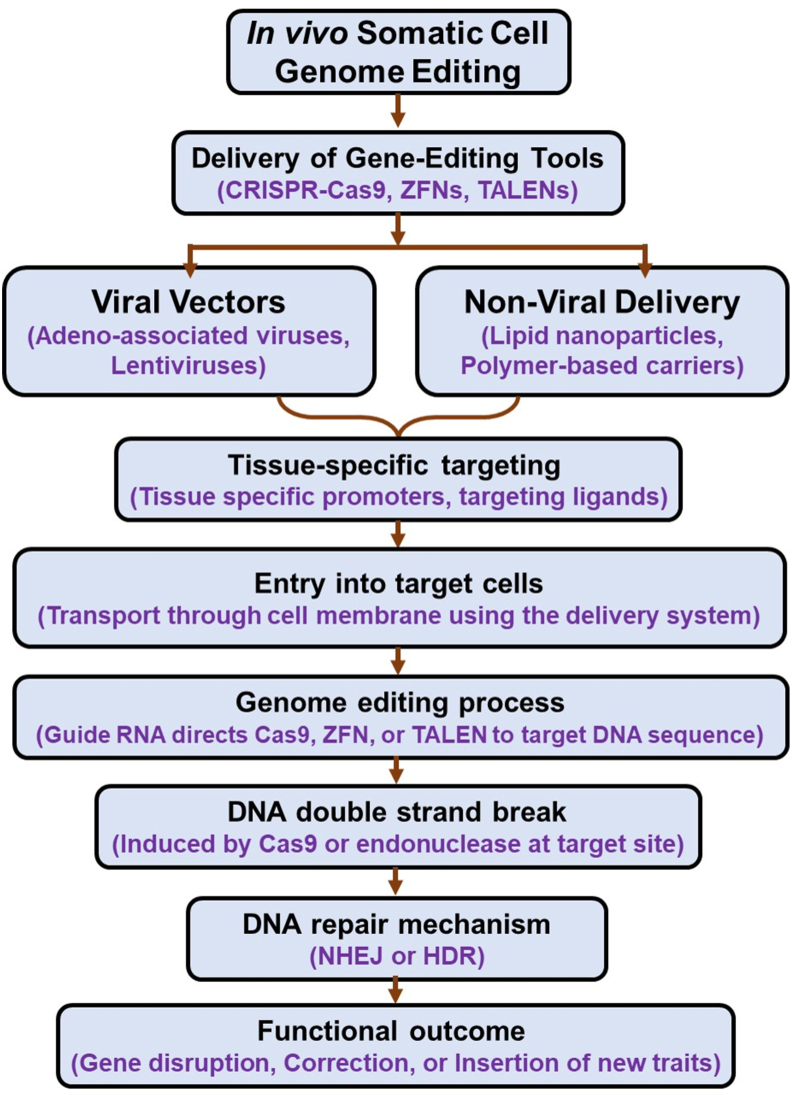
Flow chart of the mechanism of *in vivo* somatic cell genome editing.

*In vivo* editing involves the direct delivery of gene-editing components into the patient’s body, enabling the correction of genetic errors within specific tissues or organs (Fig. 3).^71^ This method relies heavily on the efficiency and specificity of delivery vectors, which include viral systems (*e.g.*, adeno-associated viruses or lentiviruses) and non-viral carriers (*e.g.*, lipid nanoparticles or polymer-based vehicles). These vectors are often modified with tissue-specific promoters or ligands to ensure precise targeting. Once administered, the vectors transport gene-editing tools like CRISPR-Cas9 into the targeted cells. Inside the cell, the CRISPR-associated enzyme (*e.g.*, Cas9) is guided by a synthetic gRNA to a specific genomic sequence, where it introduces a double-strand break. The subsequent repair process leads to the desired genetic modification, either through gene disruption via NHEJ or precise correction through HDR.^51^ *In vivo* editing has been notably used to restore dystrophin protein expression in models of Duchenne muscular dystrophy and to correct lipid metabolism genes in hepatocytes, offering potential therapeutic benefits for conditions such as familial hypercholesterolemia.^50^

**Delivery of gene-editing tools:** The gene-editing tools (CRISPR-Cas9, ZFNs, or TALENs) are delivered into the body using either viral vectors (adeno-associated viruses or lentiviruses) or non-viral systems (lipid nanoparticles, polymer-based carriers).

**Tissue-specific targeting:** Delivery vehicles are engineered to target specific tissues or cell types using tissue-specific promoters or targeting ligands.

**Entry into target cells:** Once the delivery system reaches the target tissue, it enables the gene-editing tools to enter the cells by bypassing the cell membrane.

**Genome editing process:** Inside the cell, the gRNA (in CRISPR-Cas9) or other binding elements direct the gene-editing tool (like Cas9, ZFNs, or TALEN) to the precise target DNA sequence.

**DNA double-strand break:** The endonuclease (*e.g.*, Cas9) creates a double-strand break at the targeted location on the DNA.

**DNA repair mechanism:** i) Non-homologous end joining (NHEJ): This mechanism causes insertions or deletions (indels) that disrupt faulty genes; ii) Homology-directed repair (HDR): If a donor template is provided, this repair pathway allows for precise gene correction or insertion.

**Functional outcome:** The edited gene either restores the function of the faulty gene, disrupts harmful gene activity, or introduces new genetic traits.

This technique has shown potential in correcting mutations responsible for diseases like Duchenne muscular dystrophy, where *in vivo* editing of muscle cells can restore dystrophin protein expression.^44^ Other applications include targeting liver cells to correct metabolic disorders, such as familial hypercholesterolemia, by editing genes involved in cholesterol metabolism.^51^

### *Ex vivo* somatic cell genome editing

**Figure 4:**
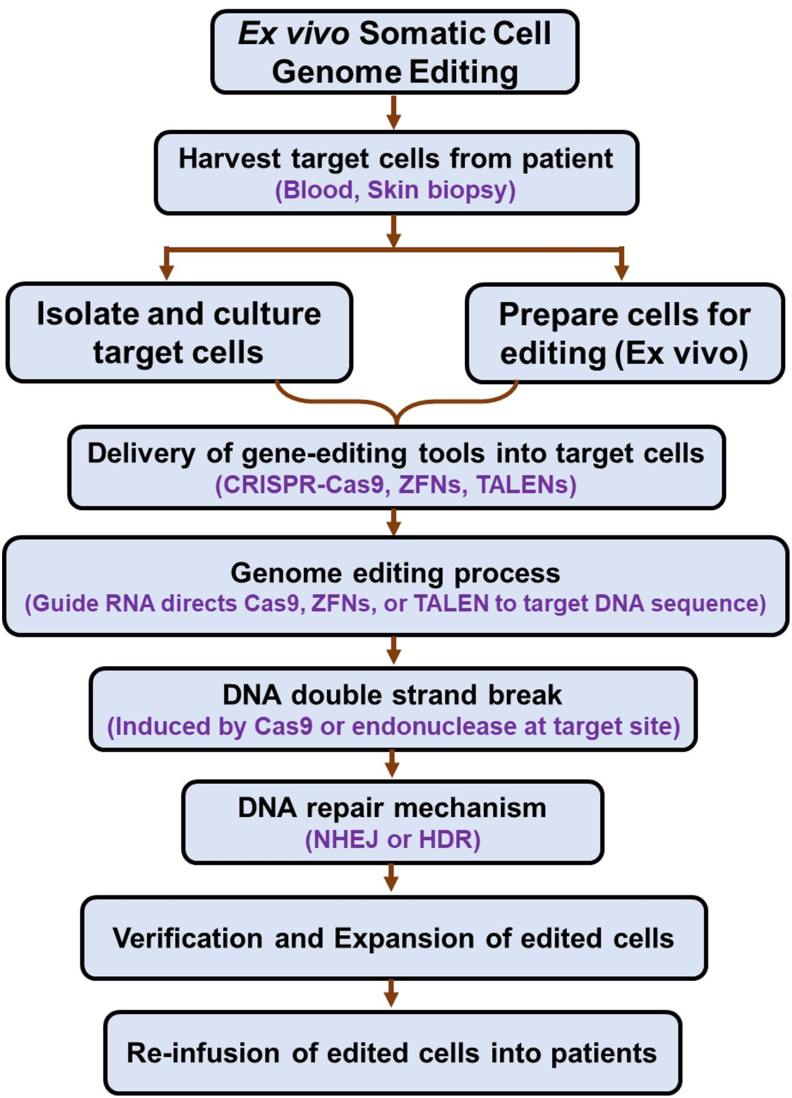
Flow chart of the mechanism of *ex vivo* somatic cell genome editing.

*Ex vivo* editing involves isolating a patient’s cells, commonly hematopoietic stem cells, T cells, or epithelial cells, followed by genetic modification in a controlled laboratory environment (Fig. 4).^72^ The edited cells are then expanded and reintroduced into the patient. This approach allows for stringent quality control, as successfully edited cells can be selected and expanded before reinfusion. Gene-editing tools are introduced into cultured cells using electroporation or viral vectors. Inside the cell, site-specific editing is achieved in a manner similar to *in vivo* approaches. The edited cells, now carrying the corrected gene or enhanced function, are reintroduced into the body where they perform their intended therapeutic role. This method has shown immense success in treating hematologic disorders, such as sickle cell disease and β-thalassemia, where editing the HBB gene in hematopoietic stem cells restores functional hemoglobin synthesis.^43^ Furthermore, chimeric antigen receptor T-cell therapy, a form of *ex vivo* editing, modifies T lymphocytes to express engineered receptors that target and destroy specific tumor antigens, revolutionizing treatment for certain leukemias and lymphomas.^73^

**Harvest target cells from patient**: Cells are collected from the patient, typically from blood or biopsied tissue, for editing *in vitro*.

**Isolate and culture target cells**: The harvested cells are isolated, cultured, and prepared for editing outside the body (*ex vivo*). This is where cells undergo growth and expansion before editing.

**Delivery of gene-editing tools**: The gene-editing tools (CRISPR-Cas9, ZFNs, TALENs) are delivered into the isolated cells using viral or non-viral delivery systems.

**Genome editing process**: The gRNA or other directing mechanisms lead the gene-editing tools to the specific DNA sequence within the target cells.

**DNA double-strand break**: The gene-editing tool (*e.g.*, Cas9) creates a double-strand break at the designated target DNA sequence.

**DNA repair mechanism**: i) Non-homologous end joining (NHEJ): Results in insertions or deletions (indels) that disrupt the gene; ii) Homology-directed repair (HDR): Involves the precise insertion or correction of genes if a donor template is provided.

**Verification and expansion of edited cells**: After editing, the cells are verified for successful editing. If successful, they are expanded to create enough edited cells for reinfusion.

**Re-infusion of edited cells into patient**: The edited cells are then reintroduced into the patient, where they will ideally perform their corrected or newly introduced function.

### Applications

Somatic genome editing has a wide range of clinical and therapeutic applications. In neuromuscular disorders like Duchenne muscular dystrophy, *in vivo* CRISPR editing has restored dystrophin production by directly modifying muscle cells.^51^ In metabolic diseases such as familial hypercholesterolemia, editing genes like PCSK9 in hepatocytes significantly reduces circulating cholesterol levels.^51^ Hematologic diseases have been among the earliest clinical successes, and editing hematopoietic stem cells *ex vivo* to correct the β-globin gene has yielded durable results in patients with sickle cell anemia and β-thalassemia.^36^ In the field of oncology, chimeric antigen receptor T-cell therapy represents a groundbreaking approach by enabling patient-derived T cells to specifically target and kill cancer cells.^73^ Beyond treatment, somatic editing also offers preventive potential: modifying immune cells to enhance resistance to HIV or editing high-risk alleles associated with hereditary cancers are under active investigation.^55^ Importantly, somatic editing technologies continue to evolve, expanding the therapeutic landscape and offering hope for conditions that previously lacked curative options. As foundational tools like CRISPR-Cas9 mature and delivery systems improve, somatic genome editing is expected to transition from proof-of-concept to widespread clinical application, setting new standards in precision medicine.^44^

### Successes

Somatic cell genome editing has demonstrated notable clinical successes, particularly in monogenic and hematologic diseases. One of the hallmark achievements is the approval of Luxturna (voretigene neparvovec) by the US FDA, which treats Leber congenital amaurosis type 2 by delivering a functional copy of the RPE65 gene using an adeno-associated virus vector. This therapy has shown clinically meaningful improvements in visual function. Another significant success is the emergence of chimeric antigen receptor T-cell therapies, such as Kymriah (tisagenlecleucel) and Yescarta (axicabtagene ciloleucel), which involve *ex vivo* editing of T cells to express chimeric antigen receptors targeting CD19 in B-cell malignancies like acute lymphoblastic leukemia and diffuse large B-cell lymphoma. These therapies have led to high remission rates and are revolutionizing immunotherapy. Collectively, these developments demonstrate the transformative potential of somatic genome editing for treating previously untreatable conditions.^38^^,^^74^

### Failures

Despite clinical progress, somatic genome editing has faced setbacks due to biological and technical limitations. One major concern is the immune response to delivery vectors, especially viral vectors such as adeno-associated viruses and lentiviruses. Pre-existing immunity or activation of the immune system can reduce treatment efficacy and pose safety risks. Additionally, off-target effects, particularly with CRISPR-Cas9 systems, can introduce unintended mutations in non-target regions of the genome, raising the risk of oncogenesis or disruption of essential genes. Another significant issue is insertional mutagenesis, a phenomenon observed in early gene therapy trials, where the integration of viral vectors into the host genome inadvertently activated oncogenes or disrupted tumor suppressor genes, resulting in leukemias.^38^ These failures underscore the importance of improving specificity, delivery safety, and long-term monitoring in gene editing therapies.

### Challenges

Somatic cell genome editing continues to face numerous technical and translational challenges. A foremost barrier is the efficient and tissue-specific delivery of genome-editing tools to target cells *in vivo*, especially for organs such as the brain, heart, or muscle, where access is limited by biological barriers. Even with advanced vectors and lipid nanoparticles, cell-type specificity and dosing remain complex. Another challenge is reducing off-target editing, which requires optimized gRNA design, high-fidelity Cas9 variants, and advanced editing tools like base editors or prime editors. Furthermore, ensuring the durability and stability of gene edits over time is essential for long-term therapeutic success. Edited cells may be cleared by the immune system or undergo epigenetic silencing, diminishing therapeutic benefits. Additionally, scaling up manufacturing processes, especially for patient-specific therapies like chimeric antigen receptor T-cell therapy, involves high costs, logistical burdens, and variability in product quality. These limitations must be addressed through interdisciplinary research and innovation before somatic genome editing can reach its full potential in clinical settings.^70^^,^^73^

### Ongoing clinical trials

Several ongoing clinical trials are pushing the boundaries of somatic genome editing across diverse disease areas. The NTLA-2001 trial (NCT04426669) is a groundbreaking study involving *in vivo* CRISPR-Cas9 editing, delivered via lipid nanoparticles, targeting the TTR gene in the liver to treat hereditary transthyretin amyloidosis. Initial results show significant reductions in circulating transthyretin protein levels, indicating therapeutic efficacy with minimal adverse effects. Another key trial, EDIT-101 (NCT03872479), focuses on Leber congenital amaurosis type 10 by correcting mutations in the CEP290 gene via subretinal injection of an adeno-associated virus-5 vector carrying CRISPR-Cas9 components. Early safety data are promising, with some patients experiencing partial visual improvement. Additionally, clinical trials are underway for sickle cell disease and beta-thalassemia, using *ex vivo* editing strategies like CRISPR and TALENs to disrupt the BCL11A gene in hematopoietic stem cells, reactivating fetal hemoglobin production. These trials represent a new era of personalized and targeted genome editing therapies that are progressing steadily through clinical pipelines.^75^^,^^76^

### Ethical considerations

Ethical considerations in somatic genome editing, while less controversial than germline editing, still require careful deliberation. Unlike germline modifications, somatic edits are non-heritable, affecting only the treated individual and thus avoiding intergenerational transmission. This makes the ethical framework for somatic editing more straightforward. However, patient safety, informed consent, and long-term monitoring remain ethical imperatives, especially in first-in-human trials involving irreversible genomic changes. A major ethical concern is equitable access to these highly expensive and resource-intensive therapies, which could exacerbate global health disparities if limited to wealthy regions or populations. Furthermore, disparities in healthcare infrastructure, particularly in low- and middle-income countries, may prevent broad implementation of genome-editing therapies. Ethical debates also include the potential misuse of somatic editing for non-therapeutic enhancement or military purposes, raising “dual-use” concerns. Therefore, robust regulatory frameworks, transparency in clinical research, and public engagement are essential to ensure responsible development and deployment of somatic genome editing technologies.^47^

### RNA-based therapies: modulating gene expression

RNA-based therapies are among the most innovative approaches in molecular medicine, focusing on modulating gene expression at the RNA level. These therapies leverage RNA molecules, such as small interfering RNA (siRNA), microRNA (miRNA), and messenger RNA (mRNA), to regulate protein synthesis and address disease mechanisms at their root. RNA interference is a natural biological process where siRNA or miRNA molecules silence specific genes by degrading their messenger RNA before translation.

## Key principles

RNA-based therapies represent a transformative approach in molecular medicine by leveraging RNA molecules to regulate gene expression and restore cellular function in disease states. The foundational steps begin with gene isolation and cloning, wherein specific gene sequences encoding therapeutic RNAs (*e.g.*, siRNA, mRNA, or antisense oligonucleotides) are identified and amplified using restriction enzymes or PCR techniques to obtain the desired DNA templates for transcription.^77^ These genes are then inserted into suitable vectors such as plasmids or bacteriophage-based systems that serve to transport and express the genetic material within host systems. The vector is selected based on factors like size compatibility, target tissue, and expression needs, and often includes regulatory elements to control transcription and stability of RNA transcripts.^78^ The process of ligation, using DNA ligase, facilitates the formation of recombinant DNA constructs that encode RNA sequences of therapeutic interest.^79^ These constructs are subsequently introduced into host cells (commonly *E. coli* or mammalian cell lines) through transformation or transfection, allowing for intracellular transcription of the therapeutic RNA. To ensure success, screening and selection are employed using markers such as antibiotic resistance or fluorescent reporters to confirm the presence and expression of the recombinant RNA constructs.^78^ Finally, under appropriate expression conditions, the encoded RNAs are transcribed and, depending on the therapeutic strategy, function through various mechanisms: mRNA-based therapies lead to translation of therapeutic proteins; siRNA and shRNA induce RNA interference and post-transcriptional gene silencing; and antisense oligonucleotides modulate pre-mRNA splicing or block translation.^79^ These RNA molecules act specifically and transiently, offering flexible and programmable solutions for modulating gene expression in cancers, genetic disorders, infectious diseases, and beyond. Overall, the precision, adaptability, and scalability of these molecular principles underpin the growing success of RNA-based therapeutic platforms in clinical research and drug development.

### Applications in medicine and pharmaceuticals

This mechanism has been harnessed to develop therapies for conditions such as hypercholesterolemia, where targeting the *PCSK9* gene significantly reduces low-density lipoprotein cholesterol levels, lowering cardiovascular disease risk.^51^^,^^80^ Similarly, RNA interference-based therapies are being explored for genetic conditions like Huntington’s disease, where mutant RNA transcripts are silenced to prevent the production of toxic proteins.^51^ mRNA-based therapeutics, which gained prominence with the success of COVID-19 vaccines, represent another frontier in disease prevention. These vaccines, such as those developed by Pfizer-BioNTech and Moderna, deliver synthetic mRNA that instructs cells to produce antigens, triggering an immune response without the risk of infection.^80^^,^^81^ Beyond infectious diseases, mRNA technology is being investigated for cancer immunotherapy, where tumor-specific antigens encoded by mRNA can stimulate the immune system to recognize and eliminate malignant cells.^59^

### Other applications

**Production of therapeutic proteins**: Recombinant human insulin (*e.g.*, Humulin), growth hormones, clotting factors (*e.g.*, factor VIII), and monoclonal antibodies.^51^

**Gene therapy**: Treating genetic disorders like cystic fibrosis and hemophilia by delivering functional genes to replace defective ones.^17^

**Vaccines**: Development of recombinant vaccines, such as the hepatitis B vaccine, by inserting viral antigens into host cells for safe immune response generation.^82^

**Diagnostics**: Production of recombinant probes and enzymes used in molecular diagnostics, including PCR-based detection of pathogens.

### Successes

RNA-based therapies have achieved remarkable success in recent years, revolutionizing both therapeutic and preventive medicine. The most prominent example is the development of mRNA-based vaccines by Pfizer-BioNTech (BNT162b2) and Moderna (mRNA-1273) for COVID-19, which demonstrated over 90% efficacy in preventing severe disease and significantly contributed to global pandemic mitigation efforts.^83^ These vaccines showed the rapid adaptability of mRNA platforms in responding to emerging infectious diseases. Another milestone is the approval of Patisiran (Onpattro), an siRNA-based therapy that treats hereditary transthyretin-mediated amyloidosis by silencing the TTR gene in the liver, effectively reducing disease-causing protein levels and improving clinical outcomes.^84^ These successes validate the clinical utility of RNA-based interventions and highlight their potential for personalized, targeted, and fast-to-develop therapeutics in both rare and widespread diseases.

### Failures

Despite these breakthroughs, RNA-based therapies are not without limitations. A major issue is the intrinsic instability of RNA molecules in biological systems; RNA is prone to degradation by nucleases, which can reduce therapeutic efficacy before reaching target cells. Furthermore, early-stage clinical trials for several RNA therapeutics in conditions such as cancer, neurological disorders, and genetic diseases have reported only modest efficacy or inconsistent results, limiting their advancement to later trial phases.^85^ In some cases, immune activation and unintended inflammatory responses have also been observed, especially with unmodified RNA constructs. These setbacks highlight the need for better RNA stabilization strategies, delivery mechanisms, and immunogenicity profiling to fully realize the promise of RNA therapeutics.

### Challenges

RNA-based therapies face several persistent scientific and logistical challenges. First, RNA molecules are inherently unstable and must be chemically modified or encapsulated to prevent degradation by extracellular and intracellular enzymes. Second, targeted delivery remains a critical hurdle. Many tissues, such as the brain or bone marrow, are difficult to reach due to biological barriers. The use of lipid nanoparticles has emerged as a key delivery strategy, offering both protection and improved uptake by cells.^64^ However, lipid nanoparticles bring their own challenges, including batch variability, potential toxicity, and high manufacturing costs, which complicate scalability. Long-term safety data are still emerging, particularly for chronic conditions requiring repeated dosing. Additionally, individual variability in RNA metabolism and immune response poses difficulties in standardizing treatments. These challenges must be addressed through continued innovation in RNA chemistry, delivery platforms, and regulatory oversight.

### Ongoing clinical trials

Numerous clinical trials are underway to expand the applications of RNA-based therapies beyond infectious diseases. The NCT04899310 trial is evaluating updated mRNA vaccines targeting emerging COVID-19 variants, including those with immune escape mutations, to ensure continued protection against SARS-CoV-2. Similarly, NCT04720534 is investigating an siRNA-based therapeutic aimed at modulating gene expression in cystic fibrosis, a genetic disorder caused by mutations in the CFTR gene.^86^^,^^87^ Additional trials are exploring mRNA therapeutics for cancer immunotherapy, autoimmune diseases, and metabolic disorders, often using personalized or tumor-specific antigen approaches. These trials underscore the broadening scope of RNA therapeutics and reflect an increasing confidence in their adaptability and efficacy in precision medicine.

### The future of DNA-based disease prevention

Emerging DNA technologies are rapidly transforming the landscape of gene modification and disease prevention. Innovative tools such as prime editing and epigenome editing are redefining precision medicine by enabling the correction of point mutations or modification of gene expression without inducing double-stranded DNA breaks. In particular, RNA-guided base editors and CRISPR-associated transposases have demonstrated the capacity to make predictable, efficient, and minimally disruptive edits at targeted genomic loci, expanding the therapeutic window for genetic disorders that were previously considered untreatable.^88^ These advancements hold profound implications for disease prevention, especially in the context of hereditary diseases, where early correction of pathogenic variants could halt disease progression before clinical onset. Additionally, CRISPR-based diagnostic platforms, such as SHERLOCK and DETECTR, offer ultra-sensitive and rapid detection of nucleic acids from pathogens and are being increasingly integrated into public health frameworks for early outbreak detection and containment, as seen during the COVID-19 pandemic.^89^

To fully realize the potential of these technologies, a well-defined roadmap for research and development is critical. This includes improving the precision and accuracy of gene editing tools, creating safer and more effective delivery mechanisms (such as lipid nanoparticles, viral vectors, or extracellular vesicles), and minimizing off-target effects that could result in unintended consequences. Additionally, it is crucial to consider the epigenetic impacts of gene editing, particularly when it comes to germline cells, as these changes can have lasting, intergenerational effects. The incorporation of AI in designing gRNAs and predicting off-target effects is emerging as an important advancement for next-generation gene editing technologies.^90^ Equally significant are the ethical, legal, and social implications surrounding human genome editing. Issues such as germline editing, consent, privacy, and equitable access to these technologies must be addressed through a collaborative approach involving geneticists, clinicians, bioethicists, and policymakers. Establishing global governance frameworks and clear regulatory standards will be crucial to ensure the responsible and ethical use of these transformative tools. If carefully developed and implemented, these technologies have the potential to shift healthcare from a reactive model to one focused on proactive, personalized disease prevention worldwide.

## Conclusion

DNA technology has emerged as a foundational element in modern medicine, facilitating precise genetic interventions for disease prevention, diagnosis, and treatment. Innovations such as CRISPR-Cas9, recombinant DNA methods, and epigenetic modifications have shifted the healthcare paradigm from reactive treatments to proactive approaches, targeting genetic disorders, chronic conditions, and infectious diseases. While challenges related to ethical concerns, safety, and equitable access persist, the continuous development of technologies like prime editing and RNA-based therapies offers significant promise for advancing precision medicine. With well-established regulatory frameworks and interdisciplinary collaboration, DNA technology is poised to revolutionize healthcare, delivering transformative benefits on a global scale.

## CRediT authorship contribution statement

**Giri Rajasekhar Dornadula:** Writing – original draft, Data curation, Conceptualization. **Chilakala Ramakrishna:** Writing – review & editing, Writing – original draft, Software, Data curation, Conceptualization. **Sadak Basha:** Writing – original draft, Data curation. **Pramod Kumar Meriga:** Writing – original draft, Data curation. **Likhitha Chintha:** Writing – original draft, Software, Data curation. **Yeshwanth Gurugari:** Writing – original draft, Software, Data curation. **Kranthi Kumar D:** Writing – original draft, Software, Data curation. **Shaik Sameena Fatima:** Software, Data curation. **Sun Hee Cheong:** Writing – review & editing, Writing – original draft, Investigation, Funding acquisition, Data curation, Conceptualization.

## Funding

This study was financially supported by University Industry Liaison of Chonnam National University (Grant number:2025-0929), and the Regional Innovation System & Education (RISE) program through the Jeollanamdo RISE Center, funded by the Ministry of Education (MOE) and the Jeollanamdo, Republic of Korea (2025-RISE-14-007).

## Conflict of interests

The authors declare no conflict of interests.
